# Effects of Intermittent Fasting on Anxiety and the Functional Connectivity of the Amygdala in Healthy Adults

**DOI:** 10.31083/AP44384

**Published:** 2025-06-30

**Authors:** Lijuan Huo, Yibo Li, Yu Fu, Zhi Yang, Lijun Jia, Chunbo Li, Bin Zhang

**Affiliations:** ^1^Institute for Brain Research and Rehabilitation, and Guangdong Key Laboratory of Mental Health and Cognitive Science, South China Normal University, 510000 Guangzhou, Guangdong, China; ^2^Key Laboratory of Brain, Cognition and Education Science, Ministry of Education, South China Normal University, 510000 Guangzhou, Guangdong, China; ^3^Department of Psychiatry, The Affiliated Brain Hospital of Guangzhou Medical University, 510000 Guangzhou, Guangdong, China; ^4^Department of Psychiatry and Psychology, College of Basic Medical Sciences, Tianjin Medical University, 300070 Tianjin, China; ^5^Institute of Mental Health, Tianjin Anding Hospital, Tianjin Medical University, 301700 Tianjin, China; ^6^Shanghai Mental Health Center, Shanghai Jiao Tong University School of Medicine, 200025 Shanghai, China; ^7^Brain Science and Technology Research Center, Shanghai Jiao Tong University, 200240 Shanghai, China; ^8^Cancer Institute, Longhua Hospital, Shanghai University of Traditional Chinese Medicine, 200032 Shanghai, China; ^9^Center for Excellence in Brain Science and Intelligence Technology (CEBSIT), Chinese Academy of Sciences, 200031 Shanghai, China

**Keywords:** intermittent fasting, anxiety, amygdala, magnetic resonance imaging, neuronal plasticity

## Abstract

**Objectives:**

This study assessed the effect of intermittent fasting on anxiety, depression, and connectivity of the amygdala by functional magnetic resonance imaging in healthy adults. The findings could provide insights into IF as a potential non-pharmacological intervention for anxiety, offering clinical significance as a cost-effective and accessible alternative or adjunct therapy.

**Methods:**

Twenty-six healthy adults followed a time-restricted eating regimen for 50 days, fasting for 18 hours daily. Assessments were conducted at baseline, during fasting (days 10, 30, and 50), and after fasting (days 20 and ~60). Measurements included body mass index (BMI), metabolic parameters, Self-Rating Anxiety Scale (SAS), Self-Rating Depression Scale (SDS), and resting-state functional magnetic resonance imaging (fMRI) connectivity of the amygdala.

**Results:**

The BMI, glucose and insulin concentrations, insulin resistance, and anxiety scores significantly decreased during and after fasting compared to the baseline measurements (all *p* < 0.05), lasting for two months. Furthermore, we used the bilateral laterobasal amygdala as seed regions, which are responsible for emotional regulation and anxiety-like behaviours; we found changes in resting-state connectivity with the postcentral gyrus on fasting days 30 and 50.

**Conclusion:**

IF reduces anxiety by modulating amygdala functional connectivity and enhancing brain plasticity, suggesting its potential as a therapeutic approach for anxiety and related emotional disorders. The findings underscore IF’s promise as an alternative or adjuvant intervention in psychiatric care.

**Clinical Trial Registration:**

The study was registered at Clinicaltrials.gov (https://www.chictr.org.cn/showproj.html?proj=136213), registration number: ChiCTR2100052473.

## Main Points

WHAT IS ALREADY KNOWN ON THIS TOPIC

∙ Intermittent fasting may be beneficial to boost mood and relieve stress, according to results of Ramadan fasting and animal studies.

WHAT THIS STUDY ADDS

∙ Using fMRI data, we found that intermittent fasting reduced anxiety levels by changing the functional connectivity of the amygdala and precentral gyrus.

HOW THIS STUDY MIGHT AFFECT RESEARCH, PRACTICE OR POLICY

∙ The beneficial effects of intermittent fasting on anxiety and human brain plasticity, suggesting that intermittent fasting has a potential to be an alternative or adjuvant treatment in psychiatric patients.

## 1. Introduction

Recently, the effect of Intermittent fasting (IF) on health has attracted attention for research. Although IF is originally performed for religious observances (e.g., Ramadan) or weight control, it has also demonstrated potential long-term benefits on longevity, psychological well-being, and brain plasticity [1]. IF is a dietary intervention that restricts the food intake period but not calories. The three overarching IF regimens are alternate-day fasting, 5:2 IF (fasting two days each week), and daily time-restricted eating (TRE). TRE has gained popularity because its well-documented benefits on metabolic diseases [2], and it is feasible for most people, allowing four-hour or more daily feeding periods.

Growing evidence indicates that IF improves mental health, especially helping boost mood and relieve stress. Animal models showed that regular IF regimens ameliorated anxiety-like behaviour [3] possibly by promoting synaptic plasticity. More importantly, randomised control trials [4] found that IF intervention decreases stress, anxiety, and depression levels without increasing fatigue. From an evolutionary perspective, IF may elicit conserved adaptive responses. For example, mood improvements during fasting may have benefitted the search for food and thus species survival. Other possible IF neurobiological mechanisms include activating the hypothalamic-pituitary-adrenal axis, reducing levels of proinflammatory cytokines, and altering leptin levels, ketone bodies, endogenous endorphins, and the serotonin system [5].

In addition, some magnetic resonance imaging (MRI) studies attempted to elucidate how a Ramadan fasting impacts human brain structure and function [6]. Blood-oxygenation-level dependent (BOLD) signals can be used to visualize brain plasticity involved in the process of fasting, showing as reorganization of neural networks or forming new connections. This is consistent with neurogenesis and the growth of synapses observed in fasting animals [7]. Yet, to date no studies have investigated how functional brain changes accompany depressive and anxious improvements following IF.

The amygdala is central to the processing of negative emotions, particularly in relation to anxiety and fear responses [8, 9]. Amygdala dysfunction is common to various mood and anxiety disorders, including post-traumatic stress disorder (PTSD), social anxiety disorder, and specific phobia [8, 10, 11]. Numerous studies have shown the abnormality in amygdala function and connectivity during both resting and negative emotional processing in patients with anxiety disorders, and even in nonclinical subjects with anxious traits [12, 13, 14]. Similarly, depression has been linked to altered amygdala connectivity [15]. Research has shown that depressed individuals exhibit decreased FC between the amygdala and regions involved in emotional processing, such as the dorsal prefrontal cortex, dorsomedial prefrontal cortex, and precuneus [16]. Furthermore, neuroimaging studies have found that amygdala dysfunction can predict treatment responses for patients with anxiety and depression [11, 13]. Nonetheless, whether and how emotional circuitry and the amygdala are involved in the benefits of IF remain unknown.

Therefore, this study aimed to investigate the effects of IF on depression, anxiety, and the associated neural processes using MRI techniques in healthy individuals. We would collect behavioural, biochemical and neuroimaging data at multiple time points, hypothesising that during and following IF, the participants show persistent improvements in anxiety and depression and alterations in the amygdala pathways.

## 2. Materials and Methods

### 2.1 Participant Recruitment and Eligibility

We recruited participants through advertisements from the communities between December 2017 and December 2018. An experienced psychiatrist interviewed the volunteers. The inclusion criteria were: aged 20–35 years; right-handed; a fasting blood glucose of 3.89–6.1 mmol/L; systolic blood pressure of 90–130 mmHg; no mental disorders based on the Diagnostic and Statistical Manual of Mental Disorders (DSM), fourth edition [17], diagnostic criteria after a Structured Clinical Interview for DSM Disorders-based interview; no severe physical disease; no pregnancy or lactation at present; no visual and hearing impairment; no history of brain injury, stroke and other organic brain diseases; no contraindications of MRI; stable weight for a year before the study; no dieting in the past year; not a night-time or shift-work worker; and not living alone or going out alone.

### 2.2 Fasting Regimens

**Fig. 1.**
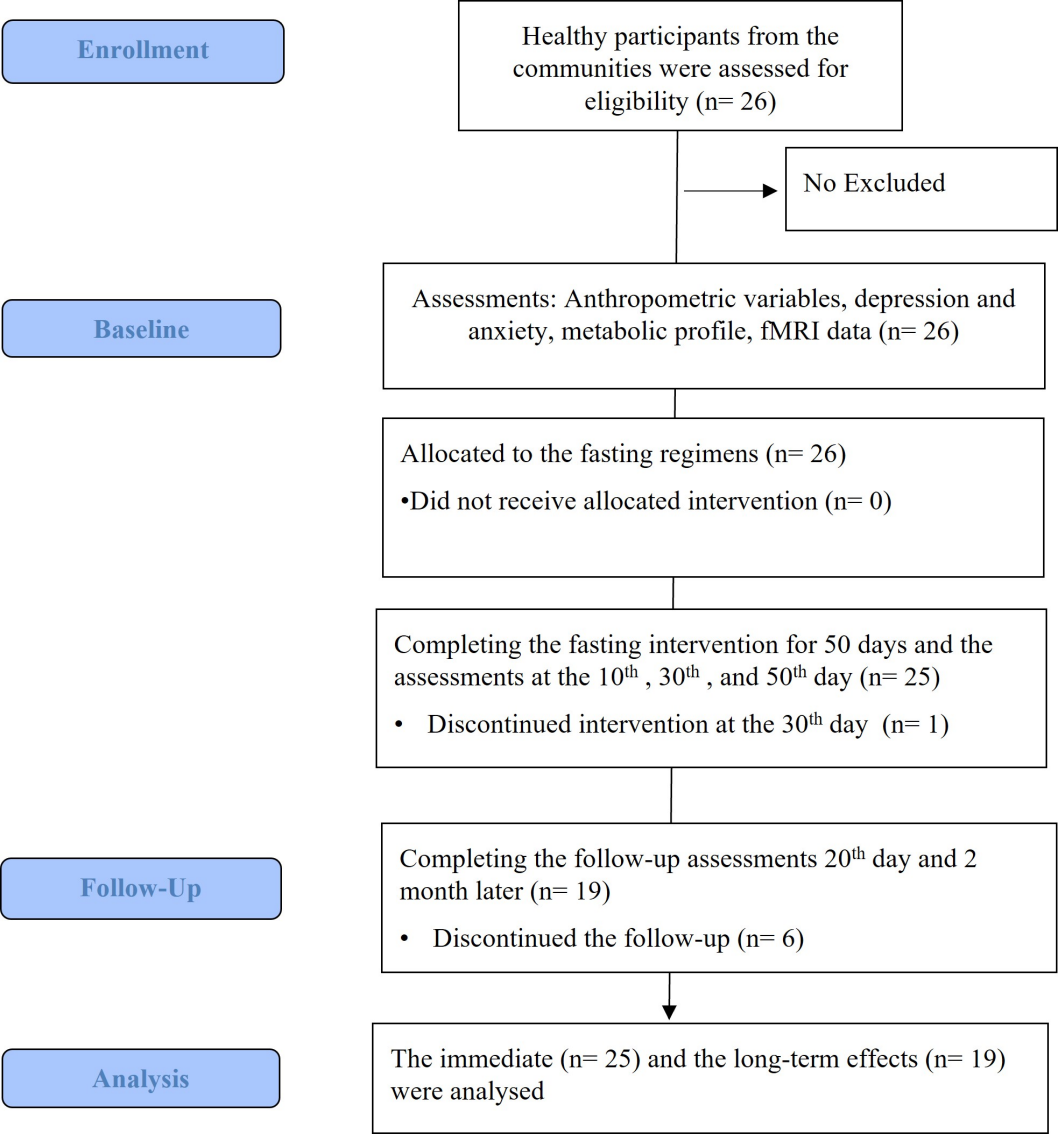
**Flow chart of the study**.

We designed a 50-day TRE regimen, which has shown benefits for both metabolic health [18] and mental health [19]. It is also the most tolerable IF option. In previous studies, the TRE regimen was usually administered for one to two months. In the TRE protocol, all participants were allowed to eat two meals between 7:30 AM and 1:30 PM (a 6-hour feeding period) but were not allowed to consume any food or beverages that included any energy (kJ) between 1:30 PM and 7:30 AM the next day (18-hour fasting period). Water consumption was acceptable during fasting. During the TRE period, participants were instructed to record the time of each meal every day. Based on these records, we excluded the participants who did not follow the fasting protocol. The TRE period lasted for 50 days, and we measured psychological, physiological, and neuroimaging data at four points: baseline (T1) and days 10 (T2), 30 (T3), and 50 (T4) of the TRE periods. Additionally, psychological and physiological data were tracked after fasting at two points: days 20 (follow-up 1, T5) and ~60 (follow-up 2, T6) after the TRE period. Fig. 1 illustrates the study design.

### 2.3 Measurements

#### 2.3.1 Anthropometric Variables

Body weight and height were assessed using standardised methods. Body weight was measured using an electronic scale calibrated to 0.1 kg (EB9003L, SENSSUN Company, Shenzhen, Guangdong, China), and the participants were weighed in light indoor clothes. Height was measured to the nearest millimetre with participants barefoot and standing upright. Body mass index (BMI) was calculated as the weight in kilograms per square of the height in meters. Based on the Working Group on Obesity in China criteria [20], BMIs of <18.5, 18.5 ≤ BMI < 24, 24 ≤ BMI < 28, and BMI ≥28 were defined as underweight, average weight, overweight, and obese, respectively.

#### 2.3.2 Blood Samples

All blood samples were collected after fasting for >6 h. The samples were immediately sent to the Laboratory Centre. The plasma glucose concentrations were analysed with an automated clinical chemistry analyser (Medica Corporation, Bedford, MA, USA), and plasma insulin concentrations were analysed using a chemiluminescence immunoassay (Siemens Healthcare Diagnostics, Deerfield, IL, USA). In addition, the homeostasis model assessment of insulin resistance was used to assess insulin resistance [21].

#### 2.3.3 Depression and Anxiety Assessments

The Self-Rating Anxiety Scale (SAS) [22] and Self-Rating Depression Scale (SDS) [23] were used to assess anxiety and depressive symptoms, respectively. The two scales used a 4-point Likert scoring method, and participants answered based on frequency: 1 = never or very rarely; 2 = occasionally; 3 = often; 4 = most of the time or always. The SAS and SDS each consist of 20 items, with total raw scores ranging from 20 to 80. Higher scores indicate more severe symptoms of anxiety or depression.

### 2.4 MRI Data Acquisition and Pre-Processing

Functional MRI (fMRI) images were acquired on a 3.0-T Siemens Verio MRI scanner (Erlangen, Germany) using an echo-planar imaging sequence with the following parameters: repetition time/echo time = 3000 ms/30 ms, field of view = 216 × 216 mm^2^, voxel size = 3 × 3 × 3 mm^3^, slice thickness = 3 mm, 45 sagittal slices, and 170 volumes. All participants were instructed to stay awake and keep their eyes closed during the resting-state data acquisition. Resting-state fMRI data were pre-processed using Statistical Parametric Mapping software (version 12, SPM12, http://www.fil.ion.ucl.ac.uk/spm). Pre-processing of fMRI data involved discarding the first 10 volumes, correcting for slice timing and head motion, performing spatial normalization to Montreal Neurological Institute space, smoothing with an 8-mm Gaussian kernel and applying band-pass filtering (0.01–0.1 Hz).

Functional connectivity (FC) analyses used seed-based linear correlation methods. The amygdala was defined as the seed region, and its subregions (laterobasal, centromedial, and superficial) were segmented using the JuBrain Cytoarchitectonic Atlas [24]. FC maps were generated by correlating time-series data between the amygdala and whole-brain regions, with statistical corrections applied for multiple comparisons (Gaussian random field corrected, *p* < 0.001, *p* < 0.05, two-tailed).

### 2.5 Statistical Analyses

All statistical analyses were conducted using SPSS 21.0 (IBM Corp., Armonk, NY, USA). All variables were tested for normality using Shapiro-Wilk Test, and non-normal variables were logarithmically transformed. The transformed variables were all normally distributed. One-way repeated-measures analysis of variance and Tukey’s HSD was conducted to assess the effects of TRE on physical, psychological and neuroimaging outcomes over the four assessment waves (baseline and days 10, 30, and 50). The long-term effects of TRE were analysed using a paired sample *t*-test. Correlations between the FC of the amygdala, SDS and SAS scores were calculated using Pearson’s correlation coefficient. All statistical tests were two-tailed, and results were considered statistically significant when *p* < 0.05.

## 3. Results

### 3.1 Participant Demographics

A total of 26 participants (mean age: 28.6 ± 3.5 years; 12 males and 14 females) were initially enrolled in the study. One participant withdrew early due to difficulty adhering to the fasting regimen, resulting in 25 participants completing the intervention phase. During the follow-up phase, six participants did not return for assessment due to scheduling conflicts or lack of interest. Among those who completed the follow-up, some questionnaire data were missing, leading to partial data loss in specific analyses. The average year of education was 15.6 ± 1.7. The group demonstrated a healthy baseline profile with average fasting glucose levels of 5.10 ± 0.38 mmol/L, BMI of 22.26 ± 2.71 kg/m^2^, systolic/diastolic blood pressure of 118/76 mmHg, fasting insulin of 67.25 ± 47.58 pmol/L. The average score of SAS and SDS were 32.16 and 31.04, respectively. Participants showed no significant differences in demographic characteristics between males and females.

### 3.2 The Effects of IF on the Anthropometric Variables, Metabolic Profile, Anxiety and Depression

IF led to significant reductions in weight management, metabolic health, and anxiety symptoms, with improvements observable as early as day 10 and sustained throughout the fasting period, as shown in Table 1. Specifically, body weight decreased progressively from 58.76 ± 9.72 kg at baseline to 54.94 ± 8.86 kg by day 50, accompanied by a significant drop in BMI from 22.26 ± 2.71 to 20.83 ± 2.55 (both *p* < 0.001). Metabolic improvements included a reduction in insulin levels (from 67.25 ± 47.58 to 39.66 ± 21.11 pmol/L, *p* = 0.033) and insulin resistance (from 2.19 ± 1.57 to 1.25 ± 0.69, *p* = 0.029), while fasting glucose levels remained stable. Psychologically, anxiety scores (SAS) decreased significantly from 32.16 ± 6.92 at baseline to 28.52 ± 6.46 on day 50 (*p* < 0.001), with no significant change in depression scores (SDS).

**Table 1.** **Changes of anthropometric variables, metabolic profile, and emotion assessments during the intermittent fasting**.

Outcome	T1	T2	T3	T4	*p*-value for ANOVA	*p*-value for Pairwise Comparisons
	Baseline	10th Day	30th Day	50th day		
Weight	58.76 ± 9.72	57.14 ± 9.61	55.66 ± 9.21	54.94 ± 8.86	<0.001	T1–T2: 0.002, T1–T3: <0.001, T1–T4: <0.001, T2–T3: 0.006, T2–T4: <0.001, T3–T4: 0.012
BMI	22.26 ± 2.71	21.65 ± 2.74	21.08 ± 2.57	20.83 ± 2.55	<0.001	T1–T2: 0.001, T1–T3: <0.001, T1–T4: <0.001, T2–T3: 0.004, T2–T4: <0.001, T3–T4: 0.015
Glucose (mmol/L)	5.10 ± 0.38	5.21 ± 0.66	5.11 ± 0.36	4.96 ± 0.39	0.220	—
Insulin^†^ (pmol/L)	54.66 (37.69, 89.14)	44.34 (23.29, 71.14)	51.17 (31.28, 70.92)	35.28 (21.48, 56.07)	0.033	T1–T4: 0.028
Insulin resistance^†^ (mU.mmol/L^2^)	1.66 (1.16, 3.06)	1.49 (0.78, 2.29)	1.72 (1.02, 2.31)	1.04 (0.69, 1.75)	0.029	T1–T4: 0.025
SAS^†^	31.00 (27.50, 34.50)	28.00 (24.50, 32.50)	30.00 (25.00, 33.50)	28.00 (24.00, 30.50)	0.001	T1–T2: 0.015, T1–T3: 0.008, T1–T4: 0.002
SDS	31.04 ± 6.37	30.20 ± 6.63	31.28 ± 8.27	31.92 ± 6.56	0.353	—

BMI, body mass index; SAS, The Self-Rating Anxiety Scale; SDS, The Self-Rating Depression Scale; ANOVA, analysis of variance. ^†^Quartiles for non-normal data. Although raw values of Insulin and Insulin resistance are presented in the table, natural log transformations were applied prior to statistical testing.

### 3.3 The Effects of IF on the FC of the Amygdala

**Fig. 2.**
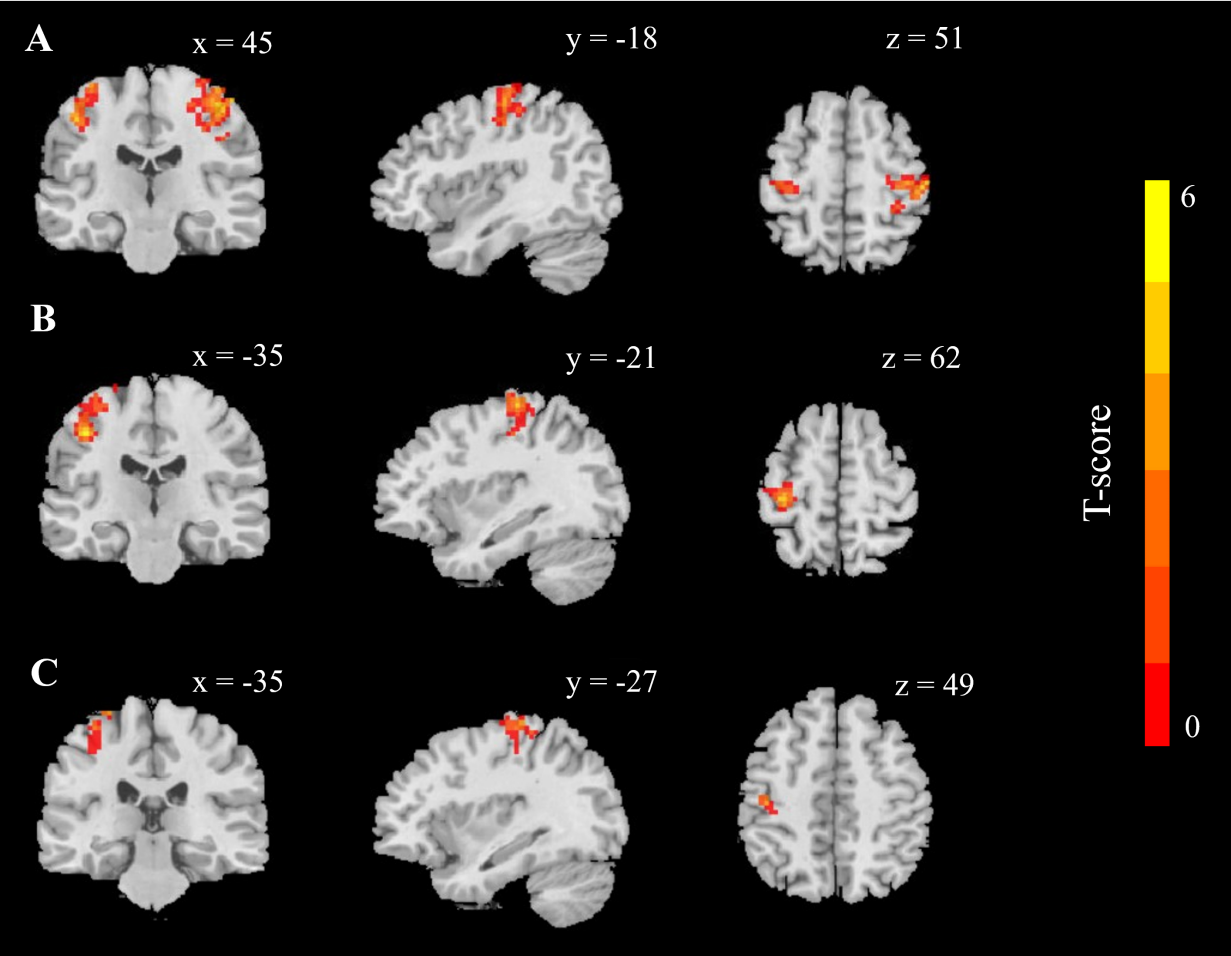
**Whole-brain functional connectivity (FC) changes of the laterobasal amygdala during intermittent fasting (Cluster-level correction for multiple comparisons was performed using Gaussian random field theory; voxel-wise *p* < 0.001, cluster-level *p* < 0.05, two-tailed)**. (A) Significant decreases in FC between the left laterobasal amygdala and the left/right postcentral gyrus on fasting day 30, relative to baseline. (B) Significant decreases in FC between the left laterobasal amygdala and left postcentral gyrus on fasting day 50, relative to baseline. (C) Significant decreases in FC between the right laterobasal amygdala and the left postcentral gyrus on fasting day 50, relative to baseline.

On fasting day 30, FC between the left laterobasal amygdala and the bilateral precentral gyrus (PG) significantly decreased compared to the baseline FC ( left PG: T = 6.01, MNI coordinates: x = 45, y = −18, z = 51; cluster size = 271 voxels; right PG: T = 5.41, x = –45, y = –21, z = 48; cluster size = 114 voxels; GRF-corrected *p* < 0.05; Fig. 2A). On fasting day 50, FC between the left laterobasal amygdala and left PG (T = 6.04, x = –42, y = –21, z = 45; cluster size = 167 voxels; GRF-corrected *p* < 0.05; Fig. 2B) and the right laterobasal amygdala and left PG (T = 5.37, x = –27, y = –30, z = 72, cluster size = 98 voxels; GRF-corrected *p* < 0.05; Fig. 2C) significantly decreased compared to the baseline FCs. The FCs of other amygdala subdivisions did not differ at any point.

Relationships were evaluated with Pearson correlation coefficients and no significant associations between changes in FC of the laterobasal amygdala and changes in SAS scores or SDS scores across all time points after multiple comparison corrections (see detailed values in the **Supplementary Table 1**).

### 3.4 The Long-Term Effects of IF

The long-term effects of IF on anthropometric and metabolic outcomes, as well as anxiety symptoms, are detailed in Tables 2,3. At the 20-day follow-up (Table 2), participants exhibited slight increases in body weight (from 54.75 ± 8.76 kg on day 50 to 55.92 ± 9.08 kg, *p* < 0.001) and BMI (from 20.41 ± 2.11 to 20.84 ± 2.23, *p* < 0.001). However, insulin levels (from 38.53 ± 22.95 to 29.78 ± 25.97 pmol/L, *p* = 0.036) and insulin resistance (from 1.26 ± 0.77 to 0.90 ± 0.85, *p* = 0.013) continued to decrease and then returned to baseline by the ~60-day follow-up. Anxiety scores remained significantly lower than baseline throughout the follow-up period (SAS: 28.11 ± 6.26 at ~60 days, *p* = 0.006).

**Table 2.** **Effects at 20 days after the end of intermittent fasting**.

Outcome	T1	T4	T5	*p*-value (T4–T1)	*p*-value (T5–T4)	*p*-value (T5–T1)
	Baseline	50th day	20th Day Post			
Weight	58.83 ± 9.73	54.75 ± 8.76	55.92 ± 9.08	<0.001	<0.001	<0.001
BMI	21.92 ± 2.29	20.41 ± 2.11	20.84 ± 2.23	<0.001	<0.001	<0.001
Insulin^†^	44.76 (29.95, 65.44)	29.09 (20.13, 58.94)	19.32 (12.34, 47.29)	0.005	0.036	0.001
Insulin resistance^†^	1.48 (0.97, 2.11)	0.84 (0.68, 1.95)	0.56 (0.40, 1.28)	0.007	0.013	<0.001
SAS^†^	31.00 (27.00, 34.00)	27.00 (24.00, 30.00)	27.00 (22.00, 33.00)	0.002	0.847	0.028
SDS	30.47 ± 6.36	30.53 ± 5.83	27.89 ± 6.07	0.966	0.012	0.011

^†^Quartiles for non-normal data. Although raw values of insulin and insulin resistance are presented in the table, natural log transformations were applied prior to statistical testing.

**Table 3.** **Effect at two months after the end of intermittent fasting**.

Outcome	T1	T4	T6	*p*-value (T4–T1)	*p*-value (T6–T4)	*p*-value (T6–T1)
	Baseline	50th day	(∼60 Days Post)			
Weight	55.83 ± 7.97	52.33 ± 7.51	53.64 ± 7.26	<0.001	0.013	0.002
BMI	21.55 ± 2.27	20.21 ± 2.36	20.71 ± 2.20	<0.001	0.013	0.002
Insulin^†^	54.70 (35.32, 68.30)	32.35 (22.51, 64.18)	33.86 (28.63, 55.93)	0.016	0.683	0.200
Insulin resistance^†^	1.74 (1.10, 2.50)	0.97 (0.69, 1.93)	1.13 (0.92, 1.84)	0.014	0.520	0.244
SAS^†^	31.00 (27.00, 34.00)	27.00 (24.00, 29.00)	27.00 (23.00, 30.00)	0.004	0.772	0.006
SDS	30.37 ± 6.05	32.32 ± 7.06	30.53 ± 8.72	0.084	0.167	0.907

^†^Quartiles for non-normal data. Although raw values of insulin and insulin resistance are presented in the table, natural log transformations were applied prior to statistical testing.

In summary, the benefits of IF on anxiety were maintained for at least two months after the fasting period, while metabolic improvements gradually returned to baseline.

## 4. Discussion

### 4.1 Summary

To our knowledge, this is the first longitudinal study investigating the neural mechanisms underlying the effects of IF on depression and anxiety in healthy individuals. We tracked how emotional status, metabolic profiles, and neural activity changed during and after IF by performing six clinical assessments and four MRI scans. We found that IF rapidly decreased the BMI, insulin concentration, insulin sensitivity, and anxiety symptoms and reduced FC between the laterobasal amygdala and PG. Moreover, such improvements in anxiety were maintained for at least two months.

### 4.2 Anxiety Reduction During and After IF

In this study, we found that in non-obese, healthy volunteers anxiety levels have decreased since the early stage of IF (i.e., day 10) and lasted for the whole process, similar to the previous pilot trial [25]. Studies from Muslim areas have also reported that Ramadan alleviated anxiety symptoms and depression [26]. Other studies on fasting interventions mainly focused on patients with diabetes or overweight/obese individuals, discovering a similar improvement in the depression and anxiety scores, general mental health, and metabolic index [27]. Despite of the substantial heterogeneity of IF studies, the available evidence suggested that IF interventions have positive and moderate impact on mental distress [28].

Notably, we used a TRE fasting regimen. Thus, daily energy intake is allowed, and there are no food restrictions, resulting in good acceptance and tolerability. In our study, only one patient dropped out during the 50 days of IF. Therefore, IF has the potential to be a novel and safe adjuvant therapy for depression and anxiety disorders. So far, no clinical study has investigated the therapeutic effect of IF in psychiatric patients, but the outcomes are promising.

Nevertheless, there are many interpretations for the efficacy of the TRE approach on anxiety, in addition to neurogenesis and synaptic plasticity mentioned in the Introduction. In many studies on Ramadan (a typical form of TRE), the mood improvements may result from religious rituals, including feelings of peace and thankfulness and lifestyle modifications (e.g., alcohol and tobacco abstinence) [4, 28]. In particular, the duration of the TRE fasting window was extended and regular. Timing of food intake in the TRE is in line with circadian rhythms. disruption of circadian rhythms induces anxiety-like behavior in gene-expression studies [29, 30]. As a result, circadian alignment enhanced by TRE may be another explanation. In the future, more studies are needed to rule out confounding factors and elucidate the mechanisms underlying TRE more clearly.

Weight loss and the feeling of weight control accompanied with IF may also contributed to mental health, which was proved by previous studies [31, 32]. Although in the current study BMI reduced following IF, we did not find a relationship between anxiety reduction and weight loss, indicating that the beneficial effects of IF on anxiety are not simply the result of successful weight management but are also due to complex brain adaptations. These processes enable neurogenesis and brain plasticity and protect neuronal networks against aberrant hyperactivity [1], which was also supported by our neuroimaging results.

### 4.3 Neural Changes in Amygdala-PG Connectivity After IF

Our findings revealed that intermittent fasting (IF) led to significant neural changes in the connectivity between the laterobasal amygdala and the postcentral gyrus (PG), a key region in the primary somatosensory cortex. Specifically, the neural coupling between the laterobasal amygdala and PG decreased after 30 and 50 days of IF. The amygdala, especially the laterobasal amygdala, plays a vital role in emotional regulation, stress-induced anxiety-like behaviors, and food-related reward mechanisms. Previous studies have implicated this brain region in food addiction and reward-seeking behaviors, emphasizing its involvement in emotional dysregulation under high-fat dietary conditions [33, 34]. Animal studies further support this, demonstrating that a high-fat diet disrupts immunoreactivity and insulin signaling in the laterobasal amygdala, thereby increasing anxiety-like behaviors [35, 36]. Our results suggest that IF may down-regulate the activity and connectivity of the laterobasal amygdala and PG, potentially alleviating anxiety.

Previous studies referring the correlation between anxiety disorders and the alteration of PG [37], where the primary somatosensory cortex is located, also supported this explanation. The role of the sensorimotor system in depression and anxiety is increasingly recognized. It is reported that stronger amygdala-PG connectivity predicts worse emotional regulation skills [38]. Connectivity in sensorimotor networks predicts depression severity and treatment response [39]. More importantly, previous studies showed that the manipulation of sensorimotor system, such as deep brain stimulation has modulatory effects on depression and anxiety, suggesting a causal relationship [40]. The reduced amygdala-PG connectivity observed in our study may reflect IF-induced changes in sensorimotor networks that mediate emotional regulation and stress responses.

It should be noted that although the IF simultaneously improved anxious symptoms and anxiety-related neural circuits, these changes did not significantly correlate in our study. The lack of correlation could be explained by the temporal dynamics of neural and psychological changes. FC may change earlier or later than symptom changes, or the relationship between FC and symptoms could be more complex than a direct, linear correlation. Alternatively, other neural, psychological, or environmental factors not captured by the current measures could be influencing the observed changes in anxiety and depression. These findings highlight the complexity of the relationship between brain connectivity and emotional symptoms. Further studies with larger sample sizes and a broader range of covariates are needed to better elucidate the intricate interactions between neural networks and psychological outcomes.

### 4.4 Limitations

Our study has some limitations. The primary limitation is the small sample size, we did not find a significant correlation between anxiety levels and decreased FC in our relatively small study population. The explanation of our findings should be considered with caution and be confirmed in a larger sample. Second, the absence of a control group made it impossible to confirm if anxiety levels and neural activity changes resulted from IF. Furthermore, we performed assessments at multiple time points during and after IF; however, we could not verify causality. Third, the clinical assessments in this study were conducted by a single experienced psychiatrist, ensuring consistency across evaluations but potentially introducing subjective bias. Future studies could use multiple evaluators, which would allow for inter-rater reliability checks, enhancing the objectivity and credibility of the clinical assessments. Fourth, there was no standardized dietary plan or guidance for participants after the 50-day fasting period. During the follow-up period, participants resumed their normal, self-determined eating habits, which may have introduced variability in outcomes and influenced the persistence of IF’s effects. Future studies could implement a post-fasting dietary protocol to minimize variability and better isolate the long-term effects of IF from dietary variations. Fifth, resting-state fMRI was this study’s only index of brain plasticity. Future studies could use multiple neuroimaging modalities, including white matter microstructure, cortical thickness, and emotional task-related activities, to analyse brain plasticity during IF.

## 5. Conclusion

This study confirms the beneficial effects of IF on anxiety and human brain plasticity, suggesting the potential therapeutic application of IF. The effectiveness of pharmacotherapy for patients with depressive or anxiety disorders is far from ideal. More than one-third of patients do not respond to drugs. Therefore, alternative or adjuvant treatment is urgently needed, and therapeutic IF is inexpensive, tolerable, and easy to perform. Thus, a randomised controlled trial with a large sample size is warranted to expand our understanding of the neural underpinnings of IF, especially in psychiatric populations.

## Data Availability

The data and materials, ethics approval, and consent to participate will be available and provided by the corresponding author if necessary.

## Supplementary Material

Supplementary material associated with this article can be found, in the online version, at https://doi.org/10.31083/AP44384.
