# Supplementary material for: Effects of Intermittent Fasting on Anxiety and the Functional Connectivity of the Amygdala in Healthy Adults
Source: Alpha Psychiatry. 2025 Jun 30;26(3):44384. doi: 10.31083/AP44384 (PMC12231414; doi:10.31083/AP44384)
Supplement: Supplementary file 1 [file 2757-8038-26-3-44384-s1.docx]

Supplemental materials

**Table S1.** Correlation coefficients between changes in the functional connectivity (FC) of the laterobasal amygdala and psychological assessments.

| **FCs** | **Amygdala-left PG** | | **Amygdala- right PG** | |
| --- | --- | --- | --- | --- |
|  | **T3-T1 (p-value)** | **T4-T1 (p-value)** | **T3-T1 (p-value)** | **T4-T1 (p-value)** |
| **SAS** | 0.079 (0.712) | -0.186 (0.384) | -0.070 (0.745) | -0.029 (0.894) |
| **SDS** | 0.434 (0.034) | 0.087 (0.684) | 0.284 (0.178) | 0.248 (0.243) |

Note: p-values were adjusted for multiple comparisons using the Bonferroni correction. Given two comparisons, the corrected significance threshold was set at p < 0.025. None of the reported p-values remained significant after correction.
